# Hypodermic Medication

**Published:** 1874-06

**Authors:** J. C. Bishop

**Affiliations:** Ohio


### Southern Medical Record.

VOL. IV.—JUNE, 1874.—NO. 6.

### Original Communications.

### HYPODERMIC MEDICATION.

BY J. C. BISHOP, M.D., OF OHIO.

*Read Before the Meigs County Medical Society, March 2, 1874.*

The day that Alexander Wood, of Edinburgh, discovered "that a solution of morphia injected under the skin, in the vicinity of a painful part, afforded remarkable relief to the pain," the clock of ages marked an epoch in scientific medicine, which indeed rivals Harvey's discovery of the circulation, or Morton's discovery of anæsthesia. At that hour, throughout the civilized world, suffering humanity became heirs to a bóon which has been to them as a pearl of price, and that particular province in the practice of medicine which looks to the alleviation of pain, which in times gone by was the bane of the physician's life, became so modified that pain presents but few phases that we may not readily overcome, and but few characters we may not subdue. Such crises in medicine as have been wrought by the discoveries of Jenner, Cinchon, Harvey, Morton, and Wood, are precursors of glorious "gala days" in their departments, and command the admiration and respect of not our profession alone, but the good and noble the world over.

Dr. Wood, after his discovery, continued to practice the method from November, 1843, to 1856, a period of thirteen years, and until he became thoroughly convinced of its utility and worth, when he published his first account of the subject in the Edinburgh *Medical and Surgical Journal*—now over thirty years ago.

Hypodermic medication, then, is a *fact*, not a fancy evanescent, arrived at the age of manhood. *Striking coincidences* seem to have existed in the early use of this method, as well as in nearly every other discovery connected with medicine; rivals for the honor of priority in discovery exist. Thus claiming priority for himself, is Mr. Rynd, of Dublin, while Dr. Kurzak, of Vienna, finds a champion in Dr. Sieveking, of London. Mr. Rynd claims that "the subcutaneous injection of medicinal substances to combat neuralgia, was first used by himself, in Meath Hospital, in 1844," at least one year subsequent to its recognized employment by Dr. Wood. Dr. Sieveking asserts that Dr. Kurzak, of Vienna, was the *first* to employ the subcutaneous or Hypodermic method, which was then largely used by Dr. Wood of Edinburgh." As yet, however, Wood has not been forestalled, and his claim to priority is generally concaded by Early and other distinguished writers on the subject, as Erlenmeyer, Lorent and Eulenberg. At the same time we are willing to accord to Wood the honor of discovery, we must allow Charles Hunter to be the author of its name.* The Hypodermic syringe was at first used for the releif of pain purely local, such as neuralgias, etc., and it seemed to Wood necessary that the puncture should be made *near* the seat of pain, at the point in which pain could be awakened by pressure. Notwithstanding Wood and his followers used the remedy locally, it is fair to presume that he was aware of the general or systematic effects, while its curative properties were ascribed to the local action of the drug so employed, and to the "affinity between the particular medicine administered and the tissue to which it is applied," must we then, if we wish to produce uterine contractions, use the remedy *locally* to obtain this effect. Charles Hunter questioned the validity of Wood's conclusions, and, by actual experiment, demonstrated that the remedy introduced beneath the skin produced its effect—which was felt equally as potent—when used in a part distant as when introduced directly in or near the painful part istelf; also that the remedy acts

*Medical Times and Gazette for March, 1859.

by *absorbtion* and *not* by *affinity for certain tissues.* His first paper on the subject appeared in the *Medical Times and Gazette*, October 16th, 1858. For a number of years the Hypodermic syringe seems to have been nearly confined to Scotland; afterwards it was taken up by Ward, Godwin, VonGräfe, Courty, Béhier, Scanzoni, Oppolzer, Lorent, Erlemneyer, Eulenberg, Fordyce Baker, Elliot, Ruppaner and others, until to-day, perhaps, there is not a country of which we ken where the blessing of freedom from pain through the potency of this method is not, to greater or less extent, felt and acknowledged. Prior to the introduction of the Hypodermic method, physicians had felt the want of some manner of modification of the oral administration of remedies. Since the stomach presents various gradations of disease, medicines coming in contact with the gastric juice, becoming mixed with other ingesta, and by the influence of conditions of inflammation, malignant disease, etc., they suffer *modification*, and some are even *entirely* changed in character, and, of course, effect. When we consider how true this proposition is, it is not wonderful that effects are sometimes produced which we had not looked for, and phenomena observed we did not care to see. On the other hand, by the method under consideration, the medicines are introduced directly into the system and absorbtion takes place at once, *without* the *slightest change* in *character*, and we *know* what we are giving, and the quantity as well—*i. e.*, if we give one-fourth of a grain of morphia, we get just the effect that one-fourth grain of that drug will produce. Given by the stomach, we *may* get the effect of one-fourth of a grain—and we *may only* get the effect of an eight of grain—a part being absorbed and the remainder carried out of the system in some other combination, the result of action of some depraved condition of the stomach.

The action of medicines given hypodermically seem, *almost* universally, to be the same, physiologically, as when given per orem, except that the effect is *greatly intensified* and more lasting. Sometimes the effect of remedies given by the syringe have seemed to me to partake of the character of shock to the nervous system, for the time impairing sensibility, and thus producing relief from pain and a temporary arrest of physiological processes—*e. g.*, the process of digestion may be completely arrested by a powerful dose of morphia, and by a *moderate* dose very materially benefited. It has seemed, also, to supply an *element* in the nervous system—a missing link, for which the economy was craving, and which seemed necessary to its physical well being, and which could not readily be supplied in any other manner. The rapidity of action when remedies are hypodermically administered is a powerful argument in its favor under many circumstances; neither is it wonderful that their effects are manifest in from two to twenty minutes, since the circulation in the human subject is *completed* once every *sixty-six* seconds, and, the medicines being thrown directly into the cellular tissue, the process of absorption *instantly* begins and *goes on* to completion. The article passes through the entire economy, and thus in contact with diseased tissues, in which its affects are manifest at once. Time and again I have seen the beneficial effects of a dose of morphia, hypodermically administered, *almost* as quickly as the point of the instrument was withdrawn. When properly used, or handled with ordinary care, the danger of producing mischief is *absolutely* nil. Improperly used, we grant, there is some danger connected with it; so, too, if any of the physical or hygienic laws are violated, danger attends, and a friendly pain admonishes us, sooner or later, that nature's laws are not to be violated with impunity. Since the hypodermic method has been practiced, two deaths *onty* are ascribed to its use, and these admit of serious questions of doubt; therefore I *infer* that, under proper restrictions, there is no safer method of administering remedial agents than this. Care is, of course, necessary to avoid puncturing a vein, but even this is a bugbear which may only frighten the inexperienced, and thus militate against its employment, to a very limited extent. This is one of the rarest of accidents. I have known the hypodermic syringe to be employed almost in every available portion of the body, and on an average of four times in twenty-four hours, for a period of several years, in the *same* patient, making an *agregate* of *six thousand and four hundred times*, without the slightest accident, and without even the formation of an abcess. The resulting abcess, of which we are reminded, is no fault of the instrument, generally, but of the solution, and the manner of its employment. This statement is made as a result of *frequent* use of the instrument, for more than four years, in my own hands. My own method of using the hypodermic syringe is as follows: After being satisfied that the instrument is in perfect order, the piston properly fitted, the barrel clean, etc., I draw up slightly more fluid than I design using, invert the syringe, press up the piston to expel the air, which is nearly always drawn in, and the surplus solution; then, with the thumb and finger of the left hand, pinch up a fold of integument, pressing it prettly firmly for a few seconds, to lessen its sensibility, with a firm, steady pressure introduce the needle, at an angle of about forty-five degree, *directly into the cellular tissue*, to the depth of from one-eight to a quarter of an inch, according to circumstances; press down the piston, withdraw the needle quickly, place a finger over the puncture to prevent the escape of fluid, and the operation is complete. The method of some physicians to introduce the needle from half to an inch or more, and make the point describe the arc of a circle, or even to withdraw it partially, before pressing in the fluid, I regard as unnecessarily painful in the first instance and superfluous in the second; and, from the extensive laceration of tissue, liable to produce supuration. Quite a number of remedial agents have been employed by this method, the most prominent among which are morphia, atropia, strychnia, quinia, ergotine, physostigma, caffein, mercury and chloroform. These have yielded the most satisfactory results, while another class, as conia, woorrara, nicotia, hydrocyanic acid, iodide of potassium, iron, arseniate, of soda, etc., have not yielded as happy results, and have fallen into disrepute in ordinary practice. The first drug employed hypodermically was morphia. And its range of application has kept pace, co-extensive with its employment. While its various salts have been used, the sulphate seems to have outgrown all others in favor, for various reasons, and is at present the favorite; it is perfectly soluble in distilled water; which is, for all purposes and under most circumstances, the very best vehicle for hypodermic use, on account of being perfectly unirritating to the the tissues and not liable to produce inflammation or abcess. Authors use solutions of different strength, but I am partial to magende's, and invariably employ it. Here I would impress the fact that when it becomes necessary to use a solution which is either strongly acid or alkaline it is a matter of necessity to render it nearly as neutral as possible; for if it partakes of either of these conditions abcess is liable to result. It has been satisfactorily demonstrated, both by observation and practice, that the *dose* of medicines, when given hypodermically, in *ordinary cases,* should not exceed from one-fourth to one-third the dose given by the stomach. At the same time caution is necessary in commencing its use, by the syringe, to "test the physological capabilities of the patient by a *moderate* dose before resorting to the *maximum* dose."* Men generally tolerate a much larger quantity than women, and in certain conditions of the latter, where there is extreme nervous excitability, the smallest dose will be found to disagree very materially with the stomach. Combined with atropia in these cases, the result is most satisfactory. The use of this medicine has heretofore been almost exclusively employed in adults; but during the past year I have known of its employment in certain convulsive disorders of children, with equally as good success as obtained in adults. When the proper dose is given, I regard it as safe for children as others. The physiological effects of morphia are too well understood to require repetition here. It was early—in fact, *first*—employed in neuralgia,† in which a *single* application cured the disease. It has yielded equally as good results in my own hands a number of times; and, although we will *often* fail to cure so promptly, by perseverence, for a limited time even, we will be satisfied with its results. At present, I cannot call to mind a *single* case of ordinary neuralgia, which I have treated hypodermically, that was not speedily and, so far as I know, *permanently* cured by a few applications. Excellent results have been achieved in the most obstinate forms of neuralgia, as sciatica.

Dr. Henry Lawson,‡ assistant physician to St. Mary's Hospital, says: "I have now accumulated over thirty cases of *true sciatica*; and, while I have found other remedies do more or less benefit to the patients, I have never found any effect a cure or remove the pain except the salts of morphia administered hypodermically and locally. They have never failed. In some instances they require to be maintained and persevered in; but they give instantaneous and effectual relief. They never impair nutrition—indeed, they promote it indirectly, and, in recent cases, they effect a cure so rapidly that the result seems almost like a charm to the pain-worm patient." My own experience, though somewhat limited in this

*Bartholow. †Wood, British Medical Journal, Aug. 28, 1858. ‡Medical Times and Gazette, Dec. 4, 1869; p. 654.

class of cases, accords, with a single exception, with that of the author quoted.

*Puerperal convulsions* have been successfully treated by the hypodermic administration of morphia. Prof. Scanzoni,* of Wurtzburg, seems to have been the first to employ it in this affection; and such authority cannot be ignored on any subject. It seems to fulfill the indications by producing quiet and sleep; after which the patient wakes without a return oi the convulsions, or, should they occur, they are greatly modified, and a continuation of the treatment soon cures the disease.

In the convulsions occurring *after labor* the morphia has been successfully emploped, in combination with Flemming's aconite—one-third of a grain of the former, with two minims of the latter—one injection being sufficient to cure the disease.† This result was obtained in two cases by this combination. Morphia seems to exert such a salutary effect on the process of digestion that it has been hypodermically employed, as a *curative* agent, in that class of dyspeptics whose nervous symptoms predominate. Certainly we would hardly expect such action when given by the stomach.‡ Dr. T. Clifford Allbut,|| of Leeds General Infirmary, has had good success with it in these cases.

In *cholera*, even in the *algidè stage*, with vomiting and dejections partaking of the character of rice water, and being incessant, a few injections seemed to cut short the disease and cure the patient.** I am, notwithstanding the high authority of Dr. Bates, inclined to believe that the recent invasion of cholera has fully demonstrated that morphia is utterly inadequate to the cure of cholera, even hypodermically; much better results have followed the use of atropia in these cases.

Charles Hunter†† demonstrated the utility of morphia in delirium tremens. He reports that in one case, two days after its first employment, his patient was discharged cured. The apathy of these patients to medicines renders their oral administration extremely difficult, and sometimes utterly impossible. If we have at

*Bull, Gen. de Therap, 1860. †Dr. R. M. Bowstead, Lancet, May 29, 1869; p. 747. ‡ This is one of the instances wherein the effects of a remedy differ when given by the different methods. || Practitioner, June, 1869; p. 342. **Bates, Lancet, Aug. 21, 1869; p. 282. ††British Medical Journal, Jan. 8, 1859; p. 19.

hand a syringe he is not *required* to swallow medicine; and, thrown into the cellular tissue, their efforts are more peimanent and decisive. I have employed the hypodermic method in this disease, and, after a single full dose, have had the satisfaction of seeing my patient asleep in half an hour, which lasted for several hours, and, on being aroused, was perfectly sane and had no return of the disease. It has been employed in this disease by Ogle, Semeleder, Lorent, Eulenberg, Ruppaner, Anstie and Bartholow, the latter of whom* very tersely gives these indications for its use: "The condition of 'horrors' or wakefulness preceding delirium, excessive and uncontrolable vomiting of food, drink and medicine. In mild cases, in which there is little tendency to depression of the vital forces, and in which the assimilation of food proceeds satisfactorily." I have employed this remedy in combination with atropia, in dypsomania, with the happiest results, where there was sleeplessness, irritability and exeessive emesis, a single injection producing sleep, from which the patient emerged refreshed and able to attend to his usual business. First employed in psychical disorders by Hunter, whose results warranted the assertion that in mania "a single dose administered beneath the skin will at once break the neck of the disease. It will often, at once, stop the delirium, correct the mental aberation and remove the exhaustion,"† while his conclusions are more or less fully verified by Mackintosh,‡ Maudsley,|| Wolff,** and Vix,†† who cured a recent case of acute mania by a slight injection of morphia. In acute cases, characterized by intense restlessness and insomnia, this method has been found exceedingly useiul.‡‡

In *cerebro spinal meningitis* the hypodermic remedy has been used, but to a limited extent, and with but indifferent results. I have no experience with it in this disease. *Coup de soliel,* or *sun stroke,* has yielded most readily to morphia subcutaneously administered. Dr. Hutchison,|||| with a single dose of one-fourth of a grain, produced instantaneous relief, which was followed by rapid recovery. It would seem necessary to discriminate between those

*Bartholow on Hypodermic Medication; p. 54, Revised Ed. †Op. Cit. ‡The subcutaneous Injection of Morphia in Insanity, 1861. ||Reynolds' System of Medicine, vol. ii., p. 60. **Archiv fiir Psychiatrie und Nervenkrankheiten, Band ii. ††Bartholow, p. 51. ‡‡Op. Cit. ||||Op. Cit., p. 57.

cases in which the encephalic substance is congested and an apoplectic condition existed, and those in which the same substance is anemic. It would be decidedly improper to administer a remedy which would increase a congestion already existing; therefore, if there appeared to be cerebral anemia, the remedy would be indicated; otherwise, not. There is no disease, scarcely excepting neuralgia, wherein the hypodermic administration of morphia has attained better results than in hysteria. It is the remedy upon which I most implicitly rely, and I have never been disappointed in thc results obtained. I doubt if a failure to relieve a patient of hysteria be *possible* if a tolerably full dose be administeied during the attack. As a remedy in *threatened abortion* it has relieved the spmptoms, and the patient been carried safely over the danger to full term. Dr. A. B. Isham,* of Cincinnati, records three cases of threatened abortion relieved in this way. Dr. John Barclay,† has employed this remedy hypodermically in *umbilical hernia,* with *obstruction of the bowels,* with great benefit; and another case of *obstructed bowels,* which resisted all other treatment for six days, was speedily relieved by this procedure.

In *disease of the heart* and *great vessels* morphia has been, for many years, thought to be contraindicated; but since the discovery of the hypodermic method it has, in the hands of some eminent physicians, risen to a position of importance in the treatment of such affections. Dr. T. Clifford Albutt‡ speaks in the following positive terms of its employment in such cases: "From small and timid beginnings I have gone forward with this marvelous remedy until I now find myself justified in using it fearlessly, in any form, and in any stage of heart and aortic disease. No matter how swollen the limbs, no matter now agitated the pulse, no matter how blue, and how turgid the face and lips, I now never hesitate to inject morphia, and scarcely ever fail, even up to the time of the dying agonies, to give relief decided enough to earn the warmest gratitude of the patient."

Gallaher|| reports a case of a physician of Pittsburg recovering from *chronic pericarditis* by the hypodermic use of morphia. In

*Am. Jour. Med. Sciences, Jan. 1873, pp. 110–111. †What Journal not remembered. ‡Practitioner, Dec. 1870, p. 342. ||N. Y. Med. Journal, vol. 13, 1871, p. 545.

that class of chronic painful affections, where death is the inevitable result, and only relief from the excruciating pain is sought, that the passage to the end may be rendered as smooth and painless as possible, the hypodermic use of morphia cannot be over-rated; while in *cancer, etc.*, it is *the great remedy for pain,** taking precedence over all others, and, I conceive, with justness. In malarious types of disease, as *congestive chills,*† its successful employment has been fairly demonstrated; while I have had perfect results from its employment in the ordinary intermittents we meet here. An injection half an hour before the expected appearance of the chill will, in the majority of cases, most effectually prevent its appearance. And when given during the paroxysm it has seemed to cut it short very decidedly. The addition of a grain or two of quinia to the injection would render the success much more satisfactory and ultimately curative. I have succeeded in relieving the *obstinate vomiting* of *pregnancy* by this method most perfectly, and, as a palliative in this condition, it is well worth a trial. To allay *muscular irritability and pain,* during surgical manipulations, where anesthesia is inadmissible, it is a valuable expedient. The employment of anesthesia is, in many cases, entirely unnecessary after an injection of morphia, where it has hitherto been employed, as in reduction of discolcations, etc.

The advantage following the hypodermic use of morphia in such affections as *chorea,* has been shown by the researches of Hunter,‡ Levick|| and others, while in *tetanus* its employment has only given negative results. The antagonism‡ of morphia to belladona and its salts is now a well authenicated fact; consequently has been extensively employed as a physiological antidote in cases of poisoning by these medicines; and the promptness of action, when hypodermically given, renders this method almost the "*sine qua non*" in their treatment, as time is everything to the patient. It has given evidence of similar antagonistic properties, to the poisoning by strychnia,** though obviously of less value than the physostigma. To Charles Hunter belongs the credit of first employing the sulphate

*Freeman, Brit. Med. Journal, June 7, 1865, p. 639. †Jos. E. Garrison, Am. Jour. Med. Sciences, April, 1870, p. 571. ‡Brit. Med. Journal, Jan. 8, 1859, p. 19. ||Am. Jour. Med. Sciences. **Allg. Med. Zeitzung, No. 80, 1869.

of atropia in the hypodermic treatment of disease. He was followed by Béhier, Coutry, Oppolzer, Lorent and others; and, as its theraputical action is less perfectly understood by the profession than morphia, the following resume of its action from Prof. Bartholow* is here. presented: "A peculiar dryness and pallor of the lower lip is the first systemic effect to be observed. The dryness quickly invades the mucous membrane of the mouth, fauces and larynx, rendering delutition somewhat difficult and the voice husky. At the same time the pupil begins to dilate, reaching its maximum dilatation in about thirty minutes. With the dilatation there occur also deranged accommodation—the vision being presbyopic—and dimness of vision, the outlines of objects being blurred and indistinct. Flushing of the face more or less deep, according to the temperament of the patient, fulness of the head, with supra-orbital pain and sense of distension and giddiness are now experienced. Increased action of the heart, and rise of bodily temperature. The pulse rises in a few minutes to nearly twice the normal number of beats, aud the thermometer exhibits elevation of temperature; but the correspondence between the pulse rate and temperature characteristic of fever do not exist. As time passes, these symptoms are increased in intensity and degree; so that when the patient sleeps hallucinations occur. Afterwards, when peristaltic movements of the intestines occur, and discharges take place, they seem to be loose. There is frequent desire to micturate, while the emission of urine is somewhat difficult, the flow taking place only after repeated trials." The dose of atropia varies from a hundred and twentieth to a forty-eigth of a grain, according to circumstances —one sixtieth being usually sufficient, especially when combined with morphia.

Its stimulating effect upon the organic nervous centres, and its opposite action on those belonging to the cerebro-spinal system, have called for its employment in a wide circle of diseases.† It has been employed in most of those affections which call for the use of a narcotic, and by some‡ it is considered superior to morphia in such cases. At the same time its curative effect seems to equal the morphia, it produces decidedly less gastric disturbance. In

*Bartholow, Hypodermic Medication. †Gallaher, N. Y. Med. Journal, 1871.
‡Brit. Med. Journal, Nov. 24, 1861, p. 554.

combination with the latter, I have never known it to produce emesis, though either used separately has, at times—the atropia much *less* frequently than the morphia—induced vomiting. The hypodermic use of atropia, in acute inflammatory affections of the brain and meninges, would appear to be contraindicated, from its tendency to promote hyperæmia of those organs; whereas, in the *chronic* form of meningitis and myalitis, its use by Lorent and others has been attended with success; based upon physiological reasons, too, if it be true that cerebral congestion follows its employment. In psychical disorders, we possess, perhaps, no better remedy in cases wherein an anemic state of the cerebral substance and vessels exist. In *neuralgia* affecting the internal viscera, the atropia is the remedy "par excellence," since it acts with peculiar efficacy in deep-seated pain, while its congenor, the morphia, wastes itself more especially on the more superficial.* It is particularly adapted to *ovarian neuralgia*, the *pain of dysmenorrhœa*, and that *following delivery*, from pressure of the uterus on nerves in the pelvic cavity. In *sciatica* its effects are said to be more curative, and at the same time more lasting than those of morphia, though not so speedily manifest. Dr. Cowdell† details five cases of sciatica, where a few injections entirely cured his patients, and urgently recommends it in rheumatism. I have occasionally employed it in combination with morphia, in the excessive pains of *acute rheumatism*, with most excellent results. It has been employed in *hydrophobia* and *tetanus*. Of its use in the former class of cases I have no experience to offer, and in the latter affection its employment has not been of any permanent benefit.‡ I should hesitate to employ it in *asthma*, where a hyperæmic condition of the cerebral vessels existed; yet, if the opposite obtained, (which I apprehend you will rarely see) its administration might be attended with benefit. I have not used the atropia in the *excessive vomiting* of *pregnancy*, yet am inclined to believe it a remedy of value. It is useful in *lead colic*, *incontinence of urine*, *dysuria*, etc., while *spermatorrhœa* has yielded to its employment, in moderate doses, administered at bed time. One of the triumphs of modern theraphy rests in the results obtained in cases of opium narcosis, by the hypodermic employ-

*Bartholow. †Med. Times and Gazette, March 17, 1860, p. 267. ‡Bartholow.

ment of atropia as an antidote. Its action is prompt and effectual, and positively certain. Patients most profoundly narcotized are soon beyond danger, if treated in this way by atropia, while, heretofore, this condition was regarded as almost universally hopeless. The employment of strychnia, hypodermically, was formerly practiced mainly through the valuable researches of Hunter, while the more recent investigations of Echeverria and Reuben A. Vance, of New York, have added materially to our fund of knowledge on this subject.

The proper vehicle for the administration of this drug seems to me to be water; while the dose ranges from one-fiftieth to one twenty-fifth of a grain, keeping in mind the peculiar tolerance of some patients, and, on the other hand, its *intolerance* by others. It is best, generally, to commence with the minimum dose, gradually increasing it until some of its effects are observed. Its physiological effects are a limited rise of temperature in the limb injected, with a feeling of warmth; a slight erection of hair follicles. In a short time there is pain in the abdomen, with sense of distentation, and borborygmi, dilatation of the pupils, cerebral pain and throbbing, giddiness ringing in the ears, feelings of dread, with flashes of light before the eyes, while the patient appears distressed and exhibits an anxious countenance. There seems but little doubt that the action of strychnia is "on the nervous system of animal and organic life," Its action, then, being directed to these nervous systems, renders it *the* remedy in certain kinds of paralysis of a local character. In these cases Dr. Reuben A. Vance* speaks in the highest terms of its remedial effect. It is indicated in *hemiplegia, paraplegia, local paralysis, progressive muscular atrophy,* and *progressive locomotor ataxia,* in which its employment is followed with gratifying results. In *gastralgia* and *cardiac neuralgia,* Dr. Anstie says "there is no such remeuy as strychnia subcutaneously injected in doses of from $\frac{1}{20}$ to $\frac{1}{60}$ of a grain." It has been used in *amaurosis* and *amblyopia* with success; and as a *tonic* hypodermically presents evidence of value. The sulphate of quina has been more or less frequently employed hypodermically, and its physiological effects are the same as when administered by the stomach,

*Journal of Pschycological Med., vol. iv., p. 367.

except that of intensification. A sufficient amount of dilute sulph. acid should be added to dissolve the salt, the menstrum being distilled water; the mixture being *filtered* to separate the particles remaining undissolved, as well as the foreign material present. The dose for hypodermic use is from one to three or four grains, according to circumstances. It has been employed by Chasseaud and McCraith*, of Smyrna, and Moore, of Bombay, in *malarial fevers* of the *pernicious type*, with success. I have fouud it efficacious in the *ordinary intermittents*, sometimes combined with morphia, sometimes alone, and have always succeeded in cutting short the chill, if given *during that period*, and of *preventing its recurrence* effectually, if administered before its expected appearance.

The recently ascribed theraputical effects of quina as an oxytocic would necessarily open up a new field for its hypodermic employment. If its action in this direction be true when administered orally, we may expect this same result, in a *much less qualified* manner, when administered subcutaneously. In *neuralgia* it has seemed to be successful, even when other remedies had failed,† as a preventive in, and as an agent possessing curative properties in *septicemia*‡ with great gastric irritability. Where a patient's life hangs by an exceedingly brittle thread, quinia has been administered hypodermically with astonishing success, and in such cases I most earnestly advise a trial.‡

In cases of *opium habit*, do doubt owing to its resemblance to the morphia, and its similar primary effects, it could be made to materially aid in the cure of this pernicious habit, and thus prolong a life otherwise rendered miserable by gratification of an appetite perverted and morbid in character. Since the recent investigations regarding the physiological effects of *physostigma*, and its employment hypodermically, the drug has taken a prominent place in the treatment of certain disorders, such as tenanus, both *traumatic* and *idiopathic*. The dose is from one-sixth to one-third of a grain of the extract dissolved in water, and rendered neutral, by the addition of an alkali. Dr. William Haining||, of Chester, in speak-

†Am. Jour. Med. Sciences, April, 1866. †Dr. T. Curtis Smith, in Kansas City Medical Journal, June, 1872, p. 159. ‡Am. Journal for January, 1874, p. 122. ||Lancet, Dec. 18, 186e, p. 834.

ing of a case of *traumatic tetanus*, in which he employed the calabar bean, says: "The antagonistic effect of the remedy upon the well marked trismus and opisthotonos was immediate and complete. The arched back, painfully tense abdomen, quivering limbs and anxious countenance, were generally relieved in from five to ten minutes after each injection, by comfortable decubitus, a feeling of drowsy ease, and, during the night, by short snatches of sleep." And again he says, "From the results obtained in this case, it may not be too much to infer that in *tetanus* the administration of calabar bean should not, in any case, be limited to effect mitigation of the symptoms, but should be perseveringly continued, in doses gradually increasing in frequency as well as in magnitude, until either the tetanic spasms are completely overcome, or the physiological effects of the drug are manifested to a dangerous degree. Once having obtained complete relaxation, it seems far from improbable that, in a large proportion of cases, careful, free repetition of the doses on the least appearance of spasm, may enable us to maintain complete control over the symptoms until their cause becomes much enfeebled, or is altogether removed." Among the physiological effects of calabar bean we observe contraction of the pupils, a feeling of tension in the eye and head, giddiness, nausea, muscular feebleness and trembling, pale and cold skin, sweating, feebleness of the pulse, vomiting and catharsis.*

We are indebted to Dr. Fraser for the following facts regarding its action: "It destroys the sensibility of the motor nerves, but does not affect the irritability of muscle. It hightens rather than diminishes the sensibility of the sensory nerves. It acts on the spinal cord, destroying its reflex power. It does not affect the brain—consciousness in animals and the intellect in man remaining unimpaired. It enfeebles the action of the heart, diminishes the number of contractions, and lessens the duration of the systole. This action is exerted through the influence of the poison on the cardiac ganglia. By paralyzing the muscles of respiration, it frequently produces death by asphyxia."† Prof. Bartholow‡ indicated the *antagonism* of the calabar bean to the action of strychnia

*Bartholow, p. 134. †Barthlow. ‡Prize essay on atropia, Am. Med. Association.

in 1869. And it has manifested such *positive* and decidedly *beneficial* results in *strychnia poisoning* that it is now generally regarded as the most most efficient remedy in cases of this dangerous character. I have employed it in a case of strychnia poisoning with the effect of almost immediately producing its beneficial results. Its action in this case is such as to warrant its employment in cases of a similar character, with, I conceive, the happiest results. M. Bourneville,* house surgeon to Paris Hospital, recommends it in cases of *epilepsy* and *chorea*, having obtained benefit in these diseases by its employment. Charles Hunter, in 1859,† asks the pertinent question : "Is chloroform applicable for hypodermic injections?" and answers it by saying chloroform is a narcotic which may be injected into the cellular tissue in urgent cases; yet the employment of this drug did not seem to be generally practiced.

Prof. Bartholow,‡ of Cincinnati, inaugurated a new procedure in the use of this remedy, only during the last year, in cases of *tic duoloureux*, by its deep injection into the tissues in the neighborhood of the pain. The physiological action of chloroform hypodermically administered seem to be at first a burning pain, with anæsthesia of the parts into which the chloroform is diffused, followed by giddiness and staggering gait, with usually the occurrence of drowsiness.|| Still later Dr. J. B. Mattison** has reported a case of *trifacial neuralgia*, in a patient suffering under the opium-habit, which was successfully treated by the deep injection of chloroform, and at the same time the desire for his accustomed stimulant disappeared, and had not returned at the time of the writing of his paper—the injection having been employed some weeks before. In none of the cases reported were there any untoward symptoms manifest, and, strange to say, *not a single abcess was developed.* The number of cases in which the deep injection of chloroform has been employed up to the present time is four ; all successful. It remains to be seen whether the remarkable results obtained in these cases will attend its employment in the future, or pass from notice by its abuse, on account of its employment in cases not eligible for its application. This remedy certainly demands

*Lancet, Aug. 14, 1869; p. 233. †Med. Times and Gazette, Sept. 10; p. 253. ‡Clinic, Sept. 27, 1873; p. 145. ||Clinic, Sept. 27, 1873. **Read before Meigs & Mason's Academy of Medicine, Feb. 19, 1874, and at this date unpublished.

careful consideration at our hands. *Caffein* has not come into extensive or extended use by the hypodermic method, and my own experience with its employment by this method is limited. It is readily soluble in distilled water, and the dose ranges from one half to two grains. It has been employed in *migraine neuralgia*, and as an antidote to *opium narcosis*. In this latter affection it has yielded valuable and decided results in my own practice. Its first effects are decided drowsiness, followed by cerebral excitement. In this connection I cannot forbear noticing the recent and most novel procedure by the hypodermic method. Dr. James B. Garrison,* of DeWitt, Arkansas, reports a case of poisoning by from ten to twenty grains of morphia; and, after injecting ten grains of extract of belladonna into each arm, at once proceeded to perforate every eligible part of his body with the needle of the hypodermic syringe, and fill his cellular tissue with coffee, until a pint had been so administered; afterwards he compelled his patient to drink about a quart of very strong coffee, followed by brandy and quinine. The patient recovered; perhaps in *spite* of this "ultra-heroic treatment"?

Mercury, as a remedy for subcutaneous employment, is, since its use in *constitutional syphilis*, by Dr. Lewin,† of Berlin, slowly but steadily forcing its claims before the profession. It is not designed to discuss the value of the remedy in this disease, farther than relates to its hypodermic employment. Some of the advantages claimed for this method are the readiness with which it affects the system, and the diminutive quantity required. Different preparations have been used; thus M. Bonilhon‡ prefers the double iodide of mercury and sodium on account of its ready solubility. Scarenzio used calomel in glycerine and water, while Hill, Hebra, Sigmund and others, prefer the corrosive sublimate, which is the preparation generally employed. One forth-eighth of a grain dissolved in distilled water is a medium dose. Dr. Grumfield,|| in detailing the results of Prof. Sigmund's investigations in constitutional syphilis, states that the smallest quantity of the salt by which a cure was obtained was one and three-quarter grains; the largest quantity

*Med. and Surg. Reporter, Feb. 7, 1874. †Dr. Thos. Jas. Walker, in Brit. Med. Journal, July 10, 1869. ‡Bul. de Therap., April 15, 1869. ||Half-yearly Comp., July, 1869, p. 317.

was six and one half grains; the fewest number of injections required was thirteen; the greatest number, forty-four. These results were obtained from its employment in sixty cases.

Ergotine has latterly been employed by the hypodermic method, and its administration is certainly advantageous in cases of *profuse uterine hemorrhage*, and also in *hemorrhage* from any of the deeper *cavities*, such as occur in *hemoptysis*, *epistaxis*, *hœmaturesis* and *metrorrhagia*. The symptoms present in acute ergotism are as follows: Nausea and vomiting, abdominal pain, diarrhoea, dryness of the throat, thirst, anorexia, itching of the extremities, numbness, lassitude, vertigo, dilatation of the pupils, drowsiness, delirium and stupor, dimunition of the force and frequency of the pulse, (rarely the reverse) with tendency to syncope, and pallor and lividity of the face.* It acts on the organic nervous system, producing contraction of the muscular fibres of the artinoles, resulting in anemia of the nervous centres. It is of extreme value in conditions of *congestion of the cerebral substance and its vessels*, relieving the congestion and mitigating the pain which necessarily results from this congestion. Prof. Laugenbeck† conceived the idea of its employment in cases of *aneurism*, and employed it in two cases with material benefit. Dr. Reuben,‡ of Hamburg, employed it in two cases of *menorrhagia* which he cured. It has been successfully used in *metrorrhagia* by Laudman,|| and in *chronic metritis* by Von Swideraki,** while Prof. Hildebrandt used it with success in *fibroid tumors of the uterus*. It should be injected immediately over the tumor in aneurism; and the results up to the present date have been such as to exclude, to a great extent, the necessity of surgical interferance.†† I have thus, gentlemen, endeavored, as the committee on "Improvements in Medicine," to bring up to the present hour the literature of the hypodermic method; and, although the length of the paper is an objection to it, I could not do the subject even partial justness by saying less."

The hypodermic use of arsenic for the relief and cure of asthma is most warmly advocated by Comagys Paul,‡‡ M.D., of Philadel-

*Bartholow. †Half-yearly abstract for July, 1869. ‡Deutsche, klinik xxxii., 1869. ||Schmidt's Jaharbücher, vol. 148, p. 301. **Berliner Klinische, Wœhenschrift, Dec., 1872. ††Middleport, March 2, 1874. ‡‡American Journal, Jan., 1874, pp. 100–101.

phia, especially where other remedies have failed. Its beneficial effects are ascribed to its action on the nervous system, and particularly over the nerves supplying the smaller bronchi. He claims to have arrested excessive dyspnœa resulting from an aggravated attack of asthma almost instantly by the hypodermic use of Fowler's solution of arsenic. He does not give the dose.

